# Three IgH isotypes, IgM, IgA and IgY are expressed in Gentoo penguin and zebra finch

**DOI:** 10.1371/journal.pone.0173334

**Published:** 2017-04-12

**Authors:** Binyue Han, Yan Li, Haitang Han, Yaofeng Zhao, Qingjie Pan, Liming Ren

**Affiliations:** 1 State Key Laboratory for Agrobiotechnology, China Agricultural University, Beijing, P. R. China; 2 Key Laboratory of Animal Reproduction and Germplasm Enhancement in Universities of Shandong, Qingdao Agricultural University, Qingdao, P. R. China; California State University Fullerton, UNITED STATES

## Abstract

Previous studies on a limited number of birds suggested that the IgD-encoding gene was absent in birds. However, one of our recent studies showed that the gene was definitely expressed in the ostrich and emu. Interestingly, we also identified subclass diversification of IgM and IgY in these two birds. To better understand immunoglobulin genes in birds, in this study, we analyzed the immunoglobulin heavy chain genes in the zebra finch (*Taeniopygia guttata*) and Gentoo penguin (*Pygoscelis papua*), belonging respectively to the order Passeriformes, the most successful bird order in terms of species diversity and numbers, and Sphenisciformes, a relatively primitive avian order. Similar to the results obtained in chickens and ducks, only three genes encoding immunoglobulin heavy chain isotypes, IgM, IgA and IgY, were identified in both species. Besides, we detected a transcript encoding a short membrane-bound IgA lacking the last two CH exons in the Gentoo penguin. We did not find any evidence supporting the presence of IgD gene or subclass diversification of IgM/IgY in penguin or zebra finch. The obtained data in our study provide more insights into the immunoglobulin heavy chain genes in birds and may help to better understand the evolution of immunoglobulin genes in tetrapods.

## Introduction

As the characteristic molecules of the adaptive immune system, immunoglobulins (Igs) are only expressed in jawed vertebrates [1, 2]. IgH (immunoglobulin heavy chain) isotype is determined by the heavy chain constant genes [3]. To date, several distinct IgH isotypes, including IgM, IgD/W, IgA, IgY, IgE, and IgG, have been identified in multiple vertebrates. IgM and IgD are the first Igs expressed during B cell development, and coexpression of both Igs on the surface of B cells is a mature B cell marker in mammals. Both IgM and IgD are found in most taxa of vertebrates and are believed to be the most primitive IgH isotypes [4]. IgY, expressed in birds, reptiles and amphibians, is considered a progenitor of the functional mammalian IgG and IgE, and is structurally close to IgE [5].

IgM maintained its primordial four CH-domain structure and has been found in nearly all jawed vertebrates, whereas many internal duplications and deletions have occurred in IgD, which generated a high degree of structural diversity [6–10].It is also known that IgD has been lost in some jawed vertebrates such as some mammals and birds [11–14]. The discovery of distinct IgH classes in jawed vertebrates has provided significant insight into the evolution of IgH genes, suggesting that both IgM and IgD (which are orthologous to IgW in lungfish and cartilaginous fish) are the most ancestral IgH classes [15]. In addition to the well-known Igs mentioned above, a number of specific IgH classes have been identified in various species, including IgNAR (in cartilaginous fish), IgZ/T (in teleosts), IgO (in platypus), IgP (in *Pleurodeles waltl*), and IgX and IgF (in amphibians) [8, 16–21].

Birds are highly evolved jawed vertebrates, and the expression and genomic organization of Ig genes in birds exhibit some unusual characteristics. The bursa of Fabricius is a critical immune-related organ involved in avian B cell development that is bird-specific [22, 23]. Previous studies have shown that there are only three IgH isotypes in birds (IgM, IgA and IgY) encoded by the Cμ, Cα and Cυ genes, respectively, and clear evidence has shown that the IgD-encoding gene is absent in chickens and ducks [11, 12, 24, 25]. However, in a recent study, one *δ* gene and two additional *μ* and υ genes were discovered in certain birds such as the ostrich and emu [4]. This result suggests that the common ancestors of modern birds might express multiple IgH isotypes, including IgD, as well as IgM and IgY subclasses.

Using the ostrich IgY sequence as a template, we performed a thorough genomic survey using BLAST searches against the 48 recently sequenced bird genomes. The results implied that the penguin and zebra finch might encode two IgY genes. The Gentoo penguin is a flightless aquatic bird that is widely distributed in the Southern Hemisphere and is a representative Sphenisciforme, which is a remarkable order of birds but the structure and expression of Igs have not yet been studied in detail [26]. The zebra finch, which diverged from the chicken (Galliformes) more than 100 million years ago (MYA) [27, 28], is a member of Passeriformes that possesses almost a half of birds. Therefore, to determine whether IgM and IgY subclass diversification is present in the Sphenisciformes or Passeriformes, we chose the Gentoo penguin and zebra finch as model birds. In this study, we cloned and analyzed Ig heavy chain genes in two different species, the Gentoo penguin (*Pygoscelis papua*) and zebra finch (*Taeniopygia guttata*), trying to elucidate the evolution of Ig genes in birds.

## Materials and methods

### Animals, RNA and DNA isolation, and reverse transcription

Our study was approved by the Animal Care and Use Committee of the China Agricultural University. Gentoo penguin (*Pygoscelis papua*) blood samples were obtained from Haichang Polar Ocean World in Qingdao, Shandong Province, China (36°N, 120°E). Zebra finches (*Poephila guttata*) were purchased from a local market named Guanyuan in Beijing, China (40°N, 116°E). The animals were handled in accordance with the guidelines of China Agricultural University regarding the protection of animals used for experimental and other scientific purposes. Zebra finches were killed after anesthetized excessively when we got them back to the laboratory immediately. All efforts were made to minimize suffering. Total RNA from whole blood and different tissues was extracted using the TRIzol Kit (Tiangen Biotech, Beijing, China). Reverse transcription was performed using M-MLV (Moloney murine leukemia virus) reverse transcriptase following the manufacturer’s instructions (Invitrogen, Carlsbad, CA, USA). One microgram of RNA was used for cDNA synthesis. Reverse transcription was performed in a 20 μL volume consisting of 4 μL of 5 × RT buffer, 1 μL 10mM dNTP (deoxy-triphosphatnucleotides solution), 2 μL 0.1M DTT, 1 μL RNaseOUT™ (40 U/μL), 1 μL moloney murine leukemia virus (M-MLV) (200 U/μL), and so on. All samples were incubated at 25°C for 10 min for primer annealing, then incubated at 37°C for 50 min.

### Transcriptome analysis

A total of 10 μg RNA (800 ng/μl) extracted from the whole blood of Gentoo penguins and those derived from the spleen and bursa of Fabricius of zebra finches were subjected to transcriptome sequencing. The RNA libraries were examined using an Agilent 2100 Bioanalyzer (Agilent Technologies, Palo Alto, CA, USA) and the ABI StepOnePlus Real-Time PCR System (Life Technologies, Waltham, MA, USA) and then sequenced using an Illumina HiSeq™ 2000 platform (Illumina, San Diego, CA, USA).

### 3’ RACE using JH-derived primers and cDNA library construction

Total RNA from Gentoo penguin whole blood and zebra finch spleen was reverse-transcribed using the primer NotI-d(T)_18_ (5’ AAC TGG AAG AAT TCG CGG CCG CAG GAA TTT TTT TTT TTT TTT TTT 3’). The 3’ RACE PCR parameters were as follows: 94°C for 5 min; followed by 40 cycles at 94°C for 30 s, 60°C for 30 s, 72°C for 90 s; and a final extension at 72°C for 5 min with LA-*Taq* DNA Polymerase (Takara, Dalian, Liaoning Province, China). The primers used were based on the conserved J_H_ region as follows: Gentoo penguin J_H_ GSP1 (5’ ATC GAC GCG TGG GGC AGC 3’) and GSP2 (5’ GCA GCG GGA CCT CCG TCA CCG TCT CCT C 3’); zebra finch J_H_ GSP1 (5’ ATT GAC GCC TGG GGC AGC 3’) and GSP2 (5’ ACC GTC GTC ACC GTC AGC 3’). Two PCR products were cloned into the pMD19-T vector (Takara, Dalian, Liaoning Province, China) to generate Ig cDNA mini-libraries.

### Analysis of CDR3 fragments

We used the 5’ RACE System for Rapid Amplification of cDNA Ends (Invitrogen, Carlsbad, CA, USA) to study CDR3. The RACE PCR was performed using Cμ-derived primers and total RNA derived from whole blood or the spleen. Positive PCR products were cloned and sequenced. The CDR3 length and amino acid usage were then calculated.

### Detection of IgH gene expression in different tissues in zebra finch by quantitative real-time PCR

Total RNA was isolated from various tissues (liver, spleen, lung, stomach, small intestine, large intestine, and the bursa of Fabricius) using an RNeasy Mini Kit (Qiagen, North Rhine-Westphalia, Hilden, Germany). RNA quantity and quality were assessed with a NanoDrop 2000 spectrophotometer (Thermo Fisher Scientific, Waltham, MA, USA). First-strand cDNA was synthesized using a QuantiTect Reverse Transcription Kit (Qiagen, North Rhine-Westphalia, Hilden, Germany) and oligo(dT)_20_ primers. Quantitative RT-PCR (qPCR) was performed using the LightCycler480 and LightCycler480 SYBR Green I Master Mix (Roche, Basel, Basel-Stadt, Switzerland) with the following cycling conditions: 95°C for 5 min; then 40 cycles at 95°C for 10 s, 60°C for 10 s, and 72°C for 10 s. Each sample was run in triplicate. The primers used were as follows: EF1A1 (sense: 5’ GCG TGA GCG TGG TAT CAC TA 3’, antisense: 5’ ACA CCA GCA GCA ACA ATC AG 3’); IGHM (sense: 5’ GGA GGT CAC CAT GAG GAA GA 3’, antisense: 5’ GAG CCA TTG GAG GAG GAT TT 3’); IGHA (sense: 5’ GGA CGA AGG GAA CAA CTT CA 3’, antisense: 5’ GTG GTC GGA GAG GAA AAG GT 3’); IGHY (sense: 5’ GGA CTC CAT CAA CGT CCA GT 3’, antisense: 5’ CAC TCT TCC CGT TCC ACT TC 3’).

### Sequence alignment and phylogenetic analysis

The accession numbers for the sequences used in the construction of phylogenetic trees in this study are presented in supplemental data **(S1 Table)**. The nurse shark IgM gene was used as an outgroup. The multiple sequence alignments and percent identity calculations were performed with ClustalW method using the MegAlign program of the LASERGENE bioinformatics computing suite (DNASTAR program). Phylogenetic trees were generated using MrBayes3.2 [29] and viewed in TreeView [30]. Multiple sequence alignments were performed using the ClustalW program [31].

## Results

### Ig isotypes expressed in the Gentoo penguin

To determine the IgH classes expressed in the Gentoo penguin, transcriptome sequencing was performed on total RNA samples isolated from whole blood. A total of 83,583,032 clean reads ranging in size from 200 bp to 500 bp were generated, accounting for 148,562 annotated unigene sequences. BLAST searches were performed against these unigenes, and three IgH partial fragments (*μ*, *α* and *υ*) were identified. Because distinct IgH isotypes can share identical JH segments, we performed 3’ RACE using JH gene segment primers and constructed a blood IgH gene library. From the 200 clones in the library, we identified membrane-bound and secretory forms of IgM and IgA and only the secretory form of IgY. We also detected a transcript encoding a short membrane-bound form of IgA (lacking the last two CH exons); however, no IgD gene was detected. The identities of these genes were verified by phylogenetic analysis.

### Ig isotypes expressed in the zebra finch

To better characterize the IgH classes expressed in the zebra finch, transcriptome sequencing was performed on total mixed RNA samples isolated from the spleen and bursa of Fabricius. A total of 55,008,340 clean reads ranging in size from 125 bp to 250 bp were generated, accounting for 43,308 annotated unigene sequences. After BLAST searches against these unigenes, we identified three IgH partial fragments (*μ*, *α* and *υ*). We performed 3’ RACE using the JH gene segment primers and RNA samples isolated from the spleen, the tissue believed to have the highest level of IgH gene expression. Consequently, we obtained the secretory form of the IgM, IgA and IgY heavy chain transcripts, similar to those observed in the Gentoo penguin; however, no membrane-bound forms of IgM, IgA or IgY were detected. The identities of these genes were verified by phylogenetic analysis.

To investigate whether the Gentoo penguin and/or the zebra finch express IgD, the degenerate primers based on the conserved Cδ regions of other species, including the ostrich, were used. Consistent with the results described above, we did not find any IgD segments.

### Characterization of the IgM CH gene

**Fig 1:**
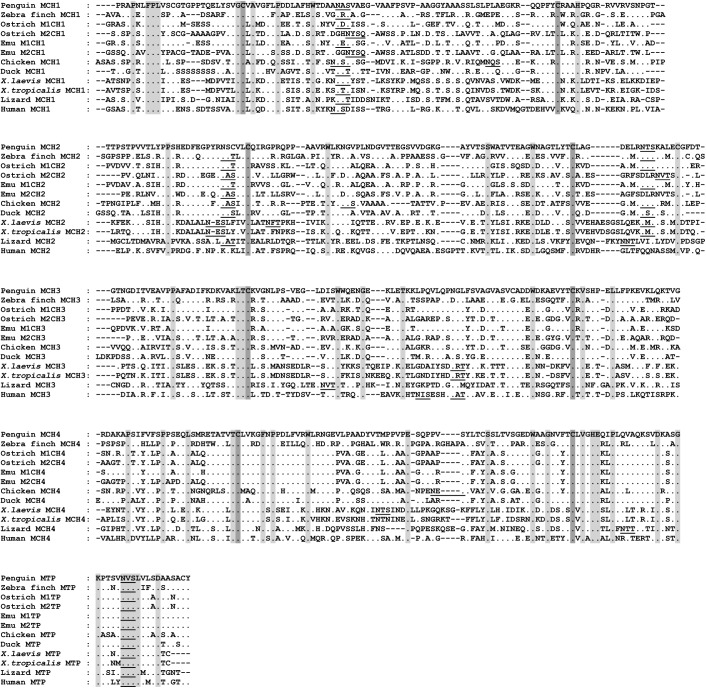
Multiple sequence alignment of the Gentoo penguin, zebra finch, ostrich, emu, chicken, duck, *X*. *laevis*, *X*. *tropicalis*, lizard and human C*μ* gene. MTP, secretory tailpiece of C*μ* gene. Identical amino acid residues were denoted as dots, and conserved amino acid residues were shaded. Canonical cysteines were marked with dark shade. Dashes were used to adjust the sequence alignment. Potential N-linked glycosylation sites were underlined. The alignment was performed using ClustalW with some manual adjustment.

**Fig 2:**
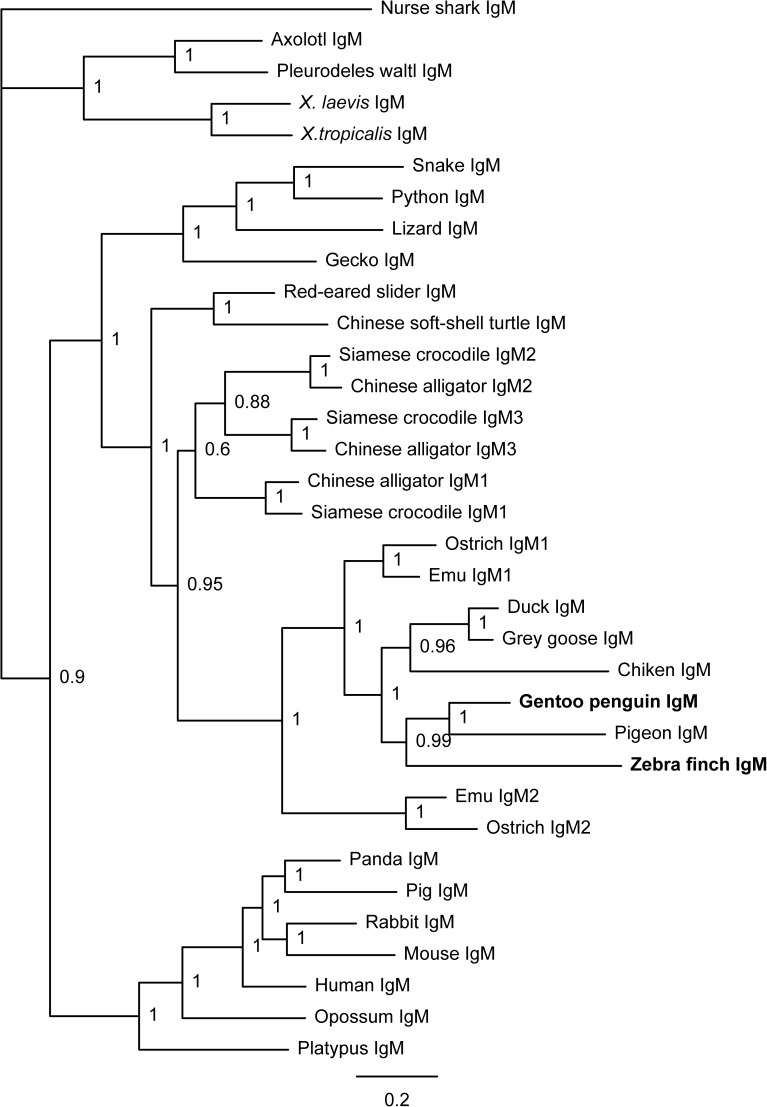
Phylogenetic analysis of IgM-encoding genes in tetrapods. Phylogenetic trees were constructed with MrBayes 3.1.2 using the amino acid sequences of the IgH constant region derived from various vertebrates. The scale bar indicates the genetic distance, and the credibility value is shown for each node. The IgM sequences of the Gentoo penguin and zebra finch were obtained in this study; all of the other sequences were obtained from NCBI GenBank (for accession numbers see the Materials and Methods section).

Similar to that in other species, the secretory form of IgM heavy chain constant region amplified from the Gentoo penguin encodes 449 amino acids consisting of four CH domains and shares 64.4% and 48.8% identity with ostrich IgM1 and ostrich IgM2, respectively. Overall, there were three potential N-linked glycosylation sites (N-X-S/T) identified throughout the entire constant region (N-45, N-199 and N-434), all of which are conserved among amphibians, reptiles, birds and humans. However, the N-127 site, which is conserved in other species including the zebra finch, was absent in the Gentoo penguin (**Fig 1**). The identities of the *μ* genes were confirmed by phylogenetic analysis (**Fig 2**).

The secretory form of the IgM heavy chain constant region from the zebra finch encodes 458 amino acids with a conserved three amino acid motif (TCY) in the carboxy-terminal, which is the site where cysteine is assumed to bind the J chain for polymer formation. The amino acid sequence of the IgM constant region shares 44.2% and 37.8% overall identity with ostrich IgM1 and ostrich IgM2, respectively. These two values are much lower than those for the Gentoo penguin, in accordance with our phylogenetic analysis. There are four conserved N-linked glycosylation sites (N-X-S/T; N-44, N-127, N-202, and N-444) in zebra finch IgM constant region, all of which are conserved among amphibians, reptiles, birds, and mammals (**Fig 2**).

Sequence alignment of the IgM constant regions of the Gentoo penguin and zebra finch with those of other species by Clustal W demonstrated that the C*μ*3 and C*μ*4 domains are less divergent than the C*μ*1 and C*μ*2 domains. We did not find any evidence indicating IgM subclass diversification in the Gentoo penguin or the zebra finch.

### Characterization of the IgA CH gene

**Fig 3:**
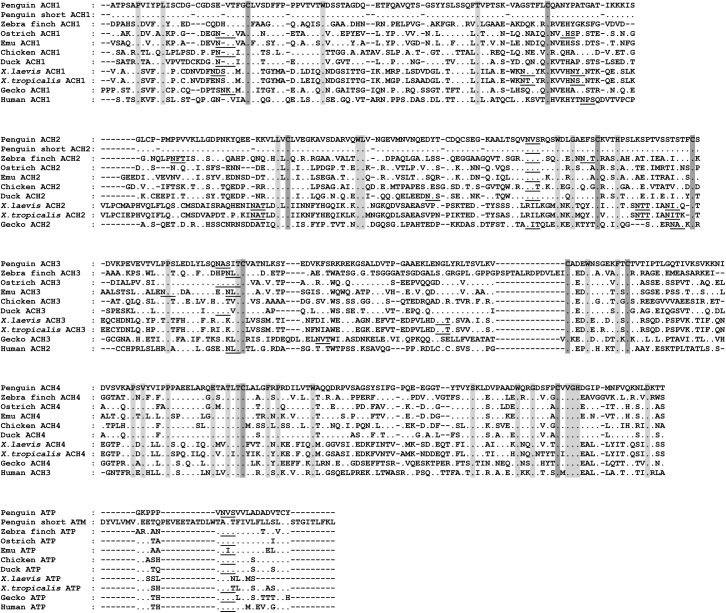
Multiple sequence alignment of the Gentoo penguin, zebra finch, ostrich, emu, chicken, duck, *X*. *laevis*, *X*. *tropicalis*, gecko, human Cα gene and Gentoo penguin short Cα gene. ATP, secretory tailpiece of Cα gene. ATM, transmembrane domain of Cα gene. Identical amino acid residues were denoted as dots, and conserved amino acid residues were shaded. Canonical cysteines were marked with dark shade. Dashes were used to adjust the sequence alignment. Potential N-linked glycosylation sites were underlined. The alignment was performed using ClustalW with some manual adjustment.

**Fig 4:**
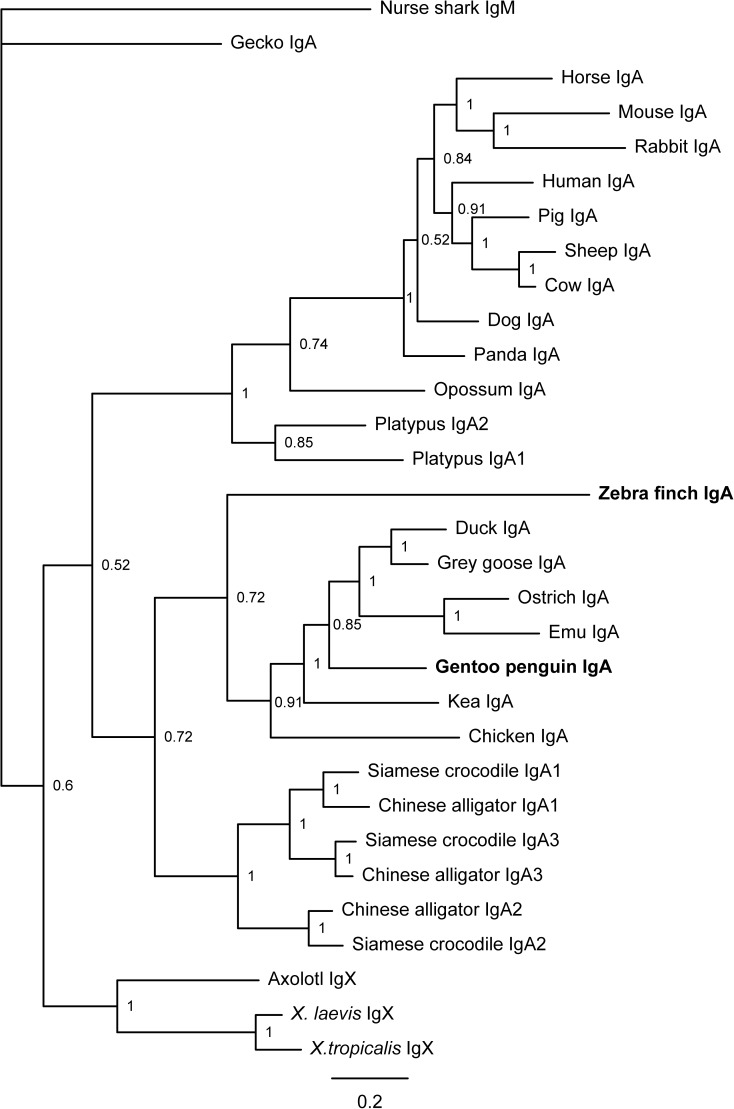
Phylogenetic analysis of IgA-encoding genes in tetrapods. Phylogenetic trees were constructed with MrBayes 3.1.2 using the amino acid sequences of the IgH constant region derived from various vertebrates. The scale bar indicates genetic distance, and the credibility value is shown for each node. The IgA sequences from the Gentoo penguin and zebra finch were obtained in this study; all of the other sequences were obtained from NCBI GenBank (for accession numbers see the Materials and Methods section).

As an important first line of defense, IgA is the principal antibody class in mucosal secretions [32]. The IgA sequences obtained from both the Gentoo penguin and zebra finch encode four CH domains. In addition, we detected a transcript encoding a short membrane-bound form of IgA (lacking the last two CH exons) in the Gentoo penguin. The short IgA consists of 252 amino acids and shares a 50.6% identity with the ostrich IgA. The four CH domains of the secretory IgA and the short membrane-bound IgA were comparatively analyzed among several birds (Gentoo penguin, zebra finch, ostrich, emu, chicken, and duck), reptile (lizard), amphibians (*X*. *tropicalis* and *X*. *laevis*) and mammal (Human) (**Fig 3**). The amino acid sequences of full length Cα gene in the Gentoo penguin and zebra finch share 56.1% and 36.6% identity with the Cα gene in ostrich, respectively. The degree of sequence identity between the Gentoo penguin and the ostrich is higher than between the zebra finch and ostrich, which agrees with the phylogenetic analysis of the IgA gene (**Fig 4**). Protein sequence alignment of the Cα genes revealed that ten conserved cysteine residues are distributed in an identical pattern between the different species. There are two N-linked glycosylation sites in CH1, CH2, and one in the IgA secretory tail, all of which are conserved in birds (**Fig 3**).

### Characterization of the IgY CH gene

**Fig 5:**
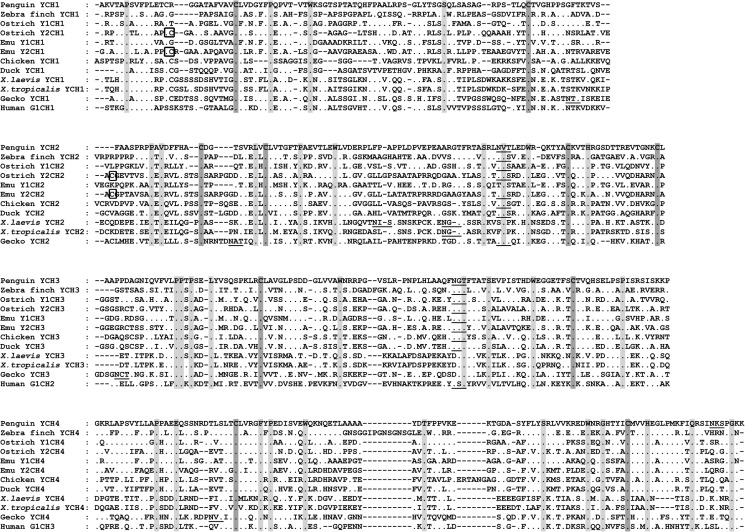
Multiple sequence alignment of the Gentoo penguin, zebra finch, ostrich, emu, chicken, duck, *X*. *laevis*, *X*. *tropicalis*, gecko C*υ* gene and human Cγ1 gene. Identical amino acid residues were denoted as dots, and conserved amino acid residues were shaded. Canonical cysteines were marked with dark shade. Dashes were used to adjust the sequence alignment. Potential N-linked glycosylation sites were underlined. The alignment was performed using ClustalW with some manual adjustment. The black boxes were used to mark the different cysteines between IgY1 and IgY2.

Initial BLAST searches against the 48 recently sequenced bird genomes showed that the penguin and zebra finch may have two IgY genes. However, only a single IgY heavy chain constant region was identified from both the Gentoo penguin and zebra finch in our study. The secretory form of IgY includes four CH domains. Compared with *υ* in the Gentoo penguin, the *υ* gene in zebra finch shows a more conserved cysteine distribution relative to IgY-encoding genes in other birds, reptiles and amphibians. Sequence alignment with other species revealed two cysteines in the C*υ*1, which suggests that these molecules associate with the light chain. Moreover, eight additional conserved cysteines were observed in C*υ*2-C*υ*4 across all of the species examined. The secretory form of the IgY constant region in the Gentoo penguin contains three potential N-linked glycosylation sites, whereas the zebra finch IgY constant region contains two potential N-linked glycosylation sites. The N-413 site is exclusive to the Gentoo penguin. Compared with IgY2 in ostrich and emu, IgY1 shows a less conserved cysteine distribution. For example, IgY2 features a conserved double cysteine in the N-terminal of CH1 and another cysteine in the N-terminal of CH2. The differences were marked with black boxes (**Fig 5**).

**Fig 6:**
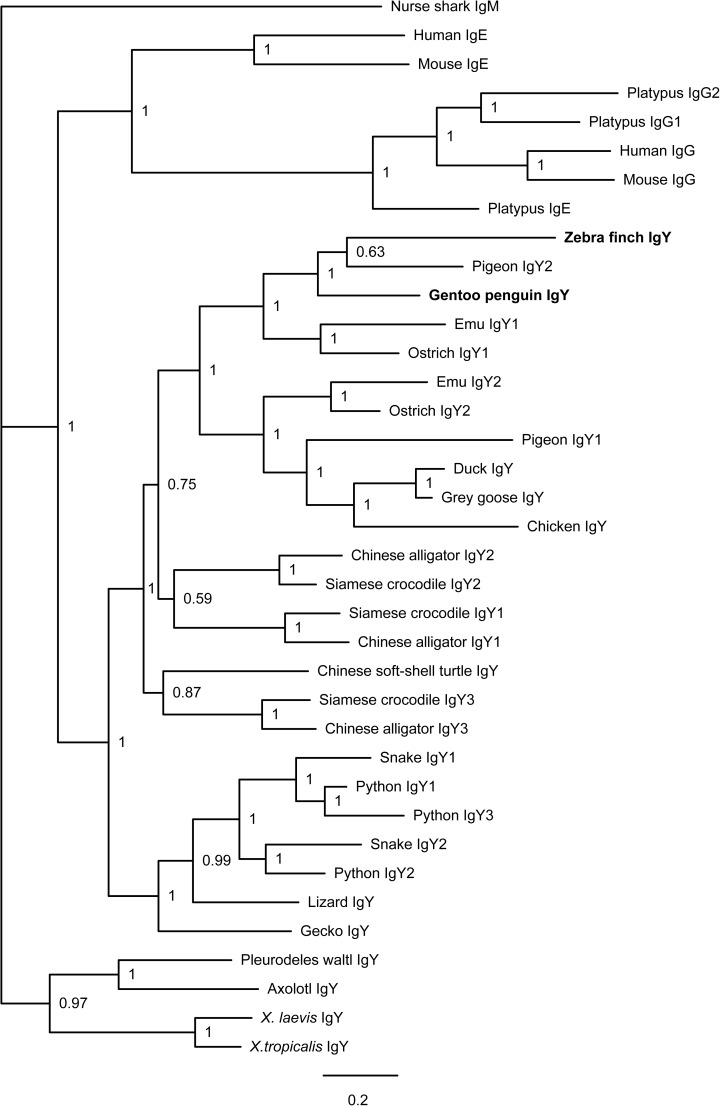
Phylogenetic analysis of IgY-encoding genes in tetrapods. Phylogenetic trees were constructed with MrBayes 3.1.2 using the amino acid sequences of the IgH constant region derived from various vertebrates. The scale bar indicates genetic distance, and the credibility value is shown for each node. The IgY sequences of the Gentoo penguin and zebra finch were obtained in this study; all of the other sequences were obtained from NCBI GenBank (for accession numbers see the Materials and Methods section).

Using the amino acid sequences of IgY-encoding genes from different jawed vertebrates, we constructed an IgY phylogenetic tree. The results suggest that subclass gene diversification likely occurred during the early stages of bird evolution but after their divergence from non-avian reptiles (**Fig 6**).

### Analysis of rearranged VDJ fragments

**Fig 7:**
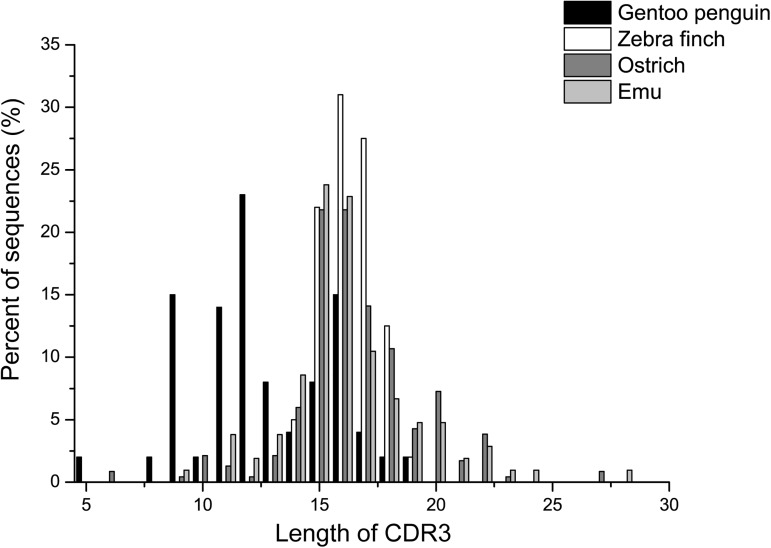
CDR3 length distribution in different species. The *x*-axis represents CDR3 length. The *y*-axis represents the relative frequency of each length.

To analyze the expressed VDJ fragments, we designed primers based on the μ gene constant region and amplified the VH regions associated with IgM heavy chains using 5’ RACE. For both the Gentoo penguin and zebra finch, all of the obtained sequences began with the same leading peptide-encoding sequence. This strongly suggests that only one VH gene can participate in VDJ recombination, and that gene conversion is the primary mechanism for antibody diversity in these species. CDR3 plays an important role in determining antibody specificity and affinity, and the length of the CDR3 region is important for the capacity to bind antigen [33]. Among the 251 clones obtained from the Gentoo penguin, 48 unique sequences were further analyzed to determine CDR3 length. The results showed that CDR3 varies from 5 to 19 amino acids, with the most common length being 12 amino acids. For the zebra finch, 200 cDNA fragments that contained 119 unique CDR3 regions were sequenced. The length of CDR3 varies from 14 to 19 residues, which is much smaller range than that of the Gentoo penguin. The length of the span is much shorter in the Gentoo penguin and zebra finch than in the ostrich and emu (**Fig 7**). There are more than two J_H_ gene segments in the Gentoo penguin and zebra finch (17 and 18, respectively).

### Quantitative real-time PCR (q-PCR) in the zebra finch

**Fig 8:**
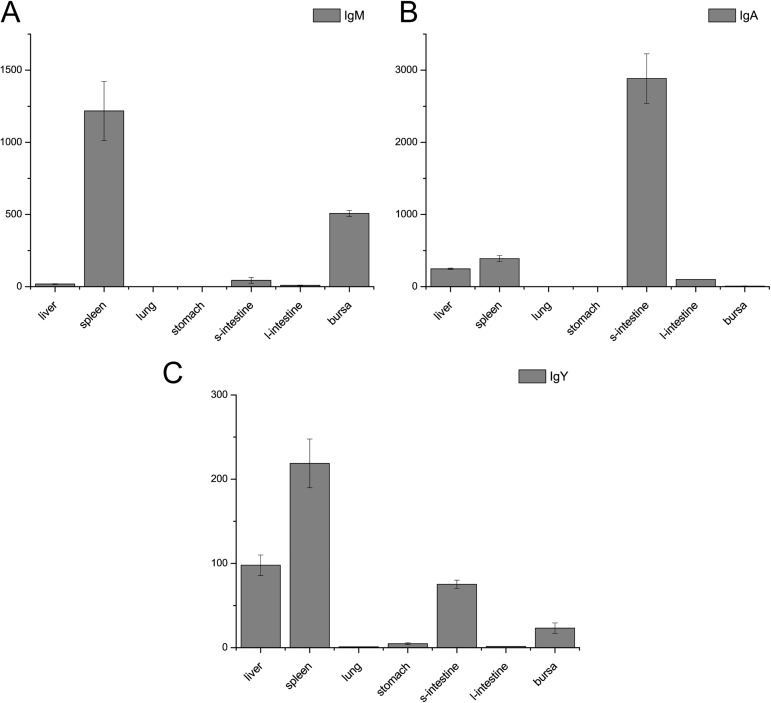
Expression of three immunoglobulin genes in the zebra finch. The data are representative of three independent experiments. The *y*-axis indicates fold changes in normalized expression. The vertical lines indicate standard deviations from the mean. The *EF1A1* gene was used as an internal control, and the values shown in the figure were calculated using the ΔΔCt method. s-intestine: small intestine; l-intestine: large intestine. (**A**) Expression of IgM in the zebra finch. (**B**) Expression of IgA in the zebra finch. (**C**) Expression of IgY in the zebra finch.

The expression pattern of immunoglobulin transcripts were examined in various tissues (liver, spleen, lung, stomach, small intestine, large intestine, and bursa of Fabricius) in the zebra finch using q-PCR. IgM is highly expressed in immune-related organs such as the spleen and the bursa of Fabricius, as expected. IgY is highly expressed in the spleen and at relatively high levels in the liver, the bursa of Fabricius and the intestine. IgA is usually highly expressed in mucosal tissue and is the main antibody class in mucosal secretions, acting as an important first line of defense [32]. In accordance with the expected results, our q-PCR data showed that zebra finch IgA is primarily expressed in the small intestine (**Fig 8**).

## Discussion

The ostrich (*Struthio camelus*) is a primitive avian species of the order Struthioniformes, having diverged from other avian lineages approximately 140 MYA [34]. Based on the recently sequenced genomes of 48 bird species and the high-throughput transcriptome sequencing of immune-related tissues, we demonstrated that the ostrich (*S*. *camelus*) possesses a functional δ gene similar to that of reptiles, such as lizards and turtles, as well as IgM and IgY subclass diversification, as in crocodilians [4,28,35]. Therefore, this study was performed for the following reasons: to determine whether IgM and IgY subclass diversification is present in the Sphenisciformes or Passeriformes and, ultimately, how widespread this phenomenon is in birds. However, in this study, only three Ig isotypes (IgM, IgA and IgY) were identified, and there was no evidence of a functional IgD gene or of subclass diversification in the Gentoo penguin or zebra finch, which is the case for most birds.

IgM subclass diversification is not common in avian reptiles (birds), although it is common in non-avian reptiles (Crocodylia, Testudines and Squamata) [36, 37]. To date, it has been reported that there are three functional *μ* genes in the crocodilian IgH locus and two in the ostrich and emu, and at least four Squamata species have more than one *μ* gene [4, 35, 38]. The isotypes mentioned above originated from duplications of the exons that encode for the C*μ* located at the 5’ end. Phylogenetic analyses suggested that the duplication in birds occurred after the divergence of Aves and reptiles and before the divergence of modern birds. Although the ostrich and emu both have two *μ* genes, many other birds, including the Gentoo penguin and zebra finch, evolutionarily retained only the *μ1* gene and lost the *μ2* gene (**Table 1**).

**Table 1:** The IgH gene isotypes in different birds.

Order	Species (references)	IgM1	IgM2	IgD	IgA	IgY1	IgY2
Struthionformes	Ostrich [4]	√	√	√	√	√	√
Casuariiformes	Emu [4]	√	√	√	√	√	√
Sphenisciformes	Penguin	√			√	√	
Passeriformes	Zebra finch	√			√	√	
Galliformes	Chicken [12]	√			√		√
Anseriformes	Duck [25]	√			√		√
Anseriformes	Goose [39]	√			√		√

As shown in the phylogenetic trees, the gene subclass diversification of *υ* genes was similar to IgM, also occurring in the early stages of bird evolution. Although the ostrich and emu, as primitive birds, encode two *υ* genes, many other birds retained only one during evolution. Unlike that almost all birds retain IgM1, our data show that some birds retain IgY1, while others retain IgY2 (**Table 1**).

In summary, based on transcriptome sequencing and additional experiments, we successfully identified three immunoglobulin heavy chain genes (*μ*, *α* and *υ*) expressed in the Gentoo penguin and zebra finch. Furthermore, a single leader peptide in the expressed heavy chain V regions indicates that gene conversion also plays a major role in the generation of antibody diversity in these two birds. Our study enriches the current knowledge of IgH gene evolution in birds and provides more data to elucidate the evolutionary history of the adaptive immune system.

## Supporting information

S1 Table The accession numbers used in the construction of phylogenetic trees. (DOCX)
