# Supplementary material for: Three IgH isotypes, IgM, IgA and IgY are expressed in Gentoo penguin and zebra finch
Source: PLoS One. 2017 Apr 12;12(4):e0173334. doi: 10.1371/journal.pone.0173334 (PMC5389807; doi:10.1371/journal.pone.0173334)
Supplement: S1 Table — (DOCX) [file pone.0173334.s001.docx]

| **μ** | **accession number** |  |  |
| --- | --- | --- | --- |
| Nurse shark IgM | I50731 | Ostrich IgM1 | AFA41927 |
| *X. laevis* IgM | AAH84123 | Ostrich IgM2 | KR030054 |
| *X. tropicalis* IgM | AAH89670 | Emu IgM1 | KU641024 |
| *Pleurodeles waltl* IgM | CAE02685 | Emu IgM2 | KU641025 |
| Axolotl IgM | A46532 | Chicken IgM | P01875 |
| Lizard IgM | ABV66128 | Duck IgM | CAC43061 |
| Gecko IgM | ABY74509 | Grey goose IgM | AFM77858 |
| Chinese soft-shell turtle IgM | ACU45376 | Gentoo penguin IgM | KX553967 |
| Python IgM | AFR33764 | Zebra finch IgM | KX553970 |
| Snake IgM | AFR33841 | Rabbit IgM | AAA64251 |
| Pigeon IgM | EMC81140 | Panda IgM | AAX73309 |
| Human IgM | AAS01769 | Platypus IgM | AAO37747 |
| Mouse IgM | CAA24199 | Opossum IgM | AF135043 |
| Pig IgM | AAC48775 | Red-eared slider IgM | AFR90255 |
| Chinese alligator IgM1 | AFZ39166 | Siamese crocodile IgM1 | AFZ39177 |
| Chinese alligator IgM2 | AFZ39167 | Siamese crocodile IgM2 | AFZ39178 |
| Chinese alligator IgM3 | AFZ39168 | Siamese crocodile IgM3 | AFZ39216 |
| **α or χ** | **accession number** |  |  |
| *X. laevis* IgX | AAI29629 | Ostrich IgA | AFA41929 |
| *X. tropicalis* IgX | AAI57651 | Emu IgA | KU641023 |
| Axolotl IgX | CAO82107 | Chicken IgA | AAB22614 |
| Gecko IgA | ABG72684 | Duck IgA | AAK17834 |
| Cow IgA | AAC98391 | Grey goose IgA | AEF79983 |
| Sheep IgA | AAC64980 | Kea IgA | Kea IgA |
| Dog IgA | AAA56796 | Gentoo penguin IgA | KX553968 |
| Opossum IgA | AAC48835 | Zebra finch IgA | KX553971 |
| Human IgA | P01876 | Panda IgA | AAX73304 |
| Mouse IgA | AAB59662 | Platypus IgA1 | AAL17700 |
| Pig IgA | I47175 | Platypus IgA2 | AAL17701 |
| Chinese alligator IgA1 | AFZ39164 | Siamese crocodile IgA1 | AFZ39174 |
| Chinese alligator IgA2 | AFZ39165 | Siamese crocodile IgA2 | AFZ39175 |
| Chinese alligator IgA3 | AFZ39173 | Siamese crocodile IgA3 | AFZ39176 |
| **υ** | **accession number** |  |  |
| *X. laevis* IgY | CAA33212 | Ostrich IgY1 | AFA41930 |
| *X. tropicalis* IgY | AAH89679 | Ostrich IgY2 | KM510516 |
| Axolotl IgY | S31436 | Emu IgY1 | KU641026 |
| Lizard IgY | ABV66132 | Emu IgY2 | KU641027 |
| Gecko IgY | ACF60236 | Chicken IgY | CAA30161 |
| Snake IgY1 | AFR33842 | Duck IgY | CAD57004 |
| Snake IgY2 | AFR33843 | Gentoo penguin IgY | KX553969 |
| Python IgY1 | AFR33765 | Zebra finch IgY | KX553972 |
| Python IgY2 | AFR33766 | Grey goose IgY | AFN08753 |
| Chinese soft-shell turtle IgY | ACU45374 | *Pleurodeles waltl* IgY | CAE02686 |
| Chinese alligator IgY1 | AFZ39169 | Siamese crocodile IgY1 | AFZ39180 |
| Chinese alligator IgY2 | AFZ39170 | Siamese crocodile IgY2 | AFZ39181 |
| Chinese alligator IgY3 | AFZ39171 | Siamese crocodile IgY3 | AFZ39182 |
| **γ** | **accession number** |  |  |
| Human IgG | AH007035 | Platypus IgG1 | AY005781 |
| Mouse IgG | CAD32497 | Platypus IgG2 | AY005782 |
| **ε** | **accession number** |  |  |
| Human IgE | AAB59395 | Platypus IgE | AY005780 |
| Mouse IgE | AAZ05128 |  |  |

**S1 Table. The accession numbers used in the construction of phylogenetic trees.**
